# Sex differences in human brain dynamics assessed with edge functional connectivity

**DOI:** 10.1007/s11682-026-01070-9

**Published:** 2026-02-12

**Authors:** Anna Rose DiPentima, Robert G. Lyday, Paul J. Laurienti, Dale Dagenbach

**Affiliations:** 1 Department of Radiology, Wake Forest School of Medicine, Winston-Salem, NC USA; 2 Department of Psychology, Wake Forest University, Winston- Salem, NC 27109 USA

**Keywords:** Sex differences, Functional connectivity, Edge connectivity, Brain network dynamics

## Abstract

Numerous sex/gender differences in the human brain have been documented, including anatomical differences and differences in structural and functional connectivity, but many of these differences are critically sensitive to methodology and many are eliminated if total brain volume is controlled for. In this study, evidence is provided for a sex/gender difference in brain network dynamics during resting state inferred from edge functional connectivity. The analyses use data from the Human Connectome Project allowing a relatively large sample size (*N* = 1024). Analyses of peak heights and trough durations in a whole brain measure of brain network dynamics based on edge functional connectivity suggest that males show less frequent alterations than females. This difference exists across multiple definitions of peak heights and trough durations, and when covaried with age and brain volume. Similar analyses using nodes within the default mode network found longer male trough durations and higher female peak heights. These results provide preliminary evidence of possible sex/gender related differences in brain dynamics when participants are in a resting state, and suggest that further studies of dynamics under other conditions may be illuminating.

## Sex differences in human brain dynamics assessed with edge functional connectivity

The neuroscience literature is replete with reports of sex/gender (s/g) differences in brain structure and function, although many of these reported differences are relatively small, come from studies with relatively small sample sizes, and have failed to survive when subjected to meta-analysis (Eliot et al., 2021). Additionally, the frequency of s/g differences may be exaggerated due to a reporting bias in which findings of differences are reported while findings of no differences are not. One recent meta-analysis of s/g differences in fMRI studies of brain activation concluded that reports of differences were subject to such reporting bias as illustrated by a lack of correlation between sample size and the number of brain foci identified as demonstrating s/g differences, and a lack of reported nonsignificant differences (David et al., 2018). Despite these issues, it is still widely accepted that s/g differences in the brain exist and identification of them remains an important goal.

## S/G structural brain differences

Studies of s/g differences in brain anatomy have produced widely variable findings and many reported differences have failed to replicate in studies with larger samples that control for brain volume. However, large scale neuroimaging studies have begun to converge on findings of greater gray matter volume (GMV) in males in the amygdala, cerebellum, putamen, and ventral occipital cortex (Liu et al., 2020). Conversely, meta-analysis controlling for brain volume found greater GMV in superior frontal and lateral parietal cortices (Ruigrok et al., 2014). One recent study using two independent large neuroimaging samples found greater GMV volumes in females in the medial and lateral prefrontal, orbitofrontal, lateral parietal, and superior temporal cortex, whereas males showed greater GMV in the ventral temporal and occipital regions, hippocampus, putamen, amygdala, and cerebellum while controlling for overall GMV (Liu et al., 2020).

### S/G functional connectivity differences

Recent interest in functional connectivity has given rise to multiple studies of S/G differences. Most commonly, studies of functional connectivity divide the brain into discrete regions (nodes) and infer activity within those regions from the blood oxygen level dependent (BOLD) functional magnetic resonance imaging (fMRI) signal. Nodes can be based on anatomical characteristics, the combination of anatomical characteristics and BOLD signal homogeneity, or even voxels. Connections between nodes, termed edges, are inferred based on the correlations of the time series of nodal BOLD signals (Rubinov & Sporns, 2010). Variations on this methodology have been used to infer the existence of approximately 8 to 10 resting state networks, including the default mode network (DMN), although again the exact characterization varies as a function of methodology (Biswal et al., 2010; Calhoun et al., 2008; Damoiseaux et al., 2006). Building on this approach, numerous s/g differences in functional brain connectivity have been reported, but lack of standard methodology makes comparisons between studies difficult. Studies vary widely in terms of how nodes are defined and the metrics used to assess connectivity. With that caution in mind, there does seem to be converging evidence from large sample studies that females show higher connectivity within the default mode network (DMN) (Biswal, 2010; Ritchie et al., 2018). In addition, other large sample studies indicate generally greater within-network connectivity in females in contrast to greater between-network connectivity in males (Allen et al., 2011; Satterthwaite et al., 2015), consistent with other structural connectivity evidence. In related work, Zhang et al. (2018) used FC to predict gender in a study that controlled for brain volume effects and found that FC in the DMN, frontoparietal, and somatosensory networks contributed the most to that prediction. Another recent large sample study that corrected for intracranial volume found greater FC in premotor, somatosensory, medial premotor cortices and the insula for males, and greater FC in inferior parietal, lateral temporal, posterior cingulate, ventromedial prefrontal, and medial temporal cortices in females (Zhang et al., 2024).

## S/G differences in node based dynamic functional connectivity

While numerous studies have examined s/g differences in functional connectivity, few have looked at s/g differences in dynamic functional connectivity. In a whole brain analysis, de Lacy et al. (2019) used an independent components analysis to identify two sets of functional intrinsic brain networks, one comprised of 24 networks and one of 51 networks, and then analyzed dynamic functional connectivity using a k-means clustering analysis in conjunction with a sliding windows approach to identify four different brain states. Females showed greater FC within the DMN than males, while males showed stronger connectivity between the DMN and a task positive control network. Females also spent more time in two of those states and switched states less frequently than males.

Another previous study examined oscillations in connectivity within the pain connectome, defined as the salience network, the default mode network, and the ascending and descending nociceptive pathways, using magnetoencephalography (MEG) data. Resting state MEG data were divided into 10 s epochs, and measures of static and dynamic functional connectivity coupling were obtained. The results suggested that males exhibited greater within network dynamic functional connectivity coupling, whereas women exhibited greater static functional connectivity coupling (Kim et al., 2021).

## Edge-Based dynamic functional connectivity

Most existing studies of dynamic connectivity employ variations of a sliding window: FC is assessed during a given temporal window which is then advanced providing a series of snapshots of FC over time (Allen et al., 2014; Hutchison et al., 2013; Mokhtari et al., 2019; Preti et al., 2016). However, despite its frequent usage, the sliding window approach has known issues including questions about the appropriate statistical analyses (Hindricks et al., 2016) and the appropriate window length (Leonardi and Van De Ville, 2015).

An alternative approach to assessing FC that can provide information about network dynamics focuses on the edges rather than the nodes in the network. Nodal FC is derived from the correlation between activity of individual neural regions. Edge FC (eFC) instead examines the cofluctuations of edges in the network which can be thought of as looking at “conversations” between pairs of nodes (Faskowitz et al., 2020). Although only a limited number of studies have employed this approach, their results suggest that it provides unique insights into brain organization. For example, while studies of community structure based on eFC produce results that resemble the communities detected based on node-centered analyses, the edge-based communities can be overlapping allowing for graded participation of particular nodes (Faskowitz et al., 2020). The brain regions that show high levels of community overlap differ from those observed with node-based analyses that also allow nodes to be assigned to multiple communities such as hierarchical modularity (Betzel et al., 2023).

Edge functional connectivity analyses begin with the creation of edge time series (ETS) data for each pair of nodes in the network. Edge time series are produced by z-scoring the time series of the BOLD signal for each node, and then, for all node pairs, calculating the element-wise products of their respective nodal time series. Values from these edge time series can in turn be correlated with each other to produce an eFC matrix (Betzel et al., 2023). In addition to being useful for creating an eFC matrix, ETS can be created and examined to determine instantaneous co-fluctuations between brain nodes, thus providing a different means of characterizing dynamic functional connectivity. Analyses of ETS find frequent fluctuations in a resting state, but also have found synchronization of co-fluctuations across participants while viewing a movie suggesting that these are relevant to the experience of the participant (Esfahlani et al., 2022). These data have proved to be useful in the exploration of various forms of individual differences such as characterizing individuals with autism spectrum disorder (ASD) (Esfahlani et al., 2022), and serving as a possible biomarker in stroke patients (Idesis et al., 2022).

Esfahlani et al. used an interesting method to summarize edge connectivity across the brain over time in a single time series: ETS data were summarized for each person using a Root Sum Square (RSS) metric. When mapped back onto the time points, this provided a single RSS time series reflecting variations in eFC across the brain over time. Esfahlani et al. then assessed differences between individuals with autism spectrum disorder and typically developing participants by comparing trough-to-trough duration, defined as the time between two consecutive trough points. On average, the duration length between two trough points was longer in the ASD participants than it was for the typically developing participants. Esfahlani et al. suggested that this might be revealing somewhat “stickier” network dynamics in ASD individuals.

Because the study of EFC may provide different insights into brain organization in communities compared to NFC, the study of edge-based dynamic functional connectivity also has the potential to new understandings. No studies to date have examined sex differences in edge-based dynamic functional connectivity. Therefore, in the current study, resting state fMRI data obtained from the Human Connectome Project (Van Essen et al., 2013) were used to compare eFC in males vs. females using RSS plots for each participant. Contrasting peak heights and trough-to-trough durations using this approach provided a unique whole brain measure of the frequency of brain reconfigurations as a function of s/g. Additional analysis using the same approach were performed using only nodes within the DMN.

## Methods

### Participants

The data for this study were taken from pre-existing Human Connectome Project (HCP) 1200 participant young connectome participants release data (Van Essen et al., 2013). After excluding participants with missing scan data, excessive motion, or preprocessing issues, minimally preprocessed fMRI resting state scans of 550 female participants and 474 male participants were acquired. Sex classification was based on the identification used in the HCP. Brain volume data for these participants were obtained from the Freesurfer data on the HCP 1200 participant release website. Participant demographics are shown in Table 1.

**Table 1:** Participant Characteristics (Mean and SE)

Sex/gender	Male	Female
N	474	550
Age	27.9 (0.11)	29.5 (0.15)
Total Brain Volume (ml)	1215.13 (44.89)	1063.80 (36.27)

### Procedures

The minimally preprocessed resting state data provided from the HCP consisted of two runs collected in a 3 T Siemens connectome-Skyra scanner, one with LR and one with RL phase encoding, with a TR of 720 ms, voxel size 2 × 2 × 2 mm, and scan time of 864 s/run (Barch et al., 2013). Ten seconds of data removed from the beginning of each run. Motion correction was performed using ICA-Aroma (Pruim et al., 2015). Bandpass filtering was performed to remove physiological noise and low frequency scanner drift. The two runs were then concatenated into a single series, and transient artifacts at the beginning and end were removed through the use of a windowed wavelet transform (Bahrami et al., 2022).

For each participant, the resulting data were then parcellated into 268 nodes based on the Shen atlas (Shen et al., 2013). The BOLD signal time series for each node was calculated and z-transformed.

Edge time series data were generated for each possible node pair by multiplying the z-transformed node time series values at each time point. The resulting 35,778 edge time series were in turn combined into a single root sum square (RSS) time series for each participant by squaring the value in each edge time series at each time point, summing those values at each time point, and then taking their square root. Matlab scripts used to perform these analyses are available at https://github.com/rlyday/LCBN_RSS.

A parallel set of analyses was done using only 51 nodes associated with the DMN in the Shen atlas (Shen et al., 2013). In those analyses, only within DMN connections were used as edges.

### Analyses

**Fig. 1:**
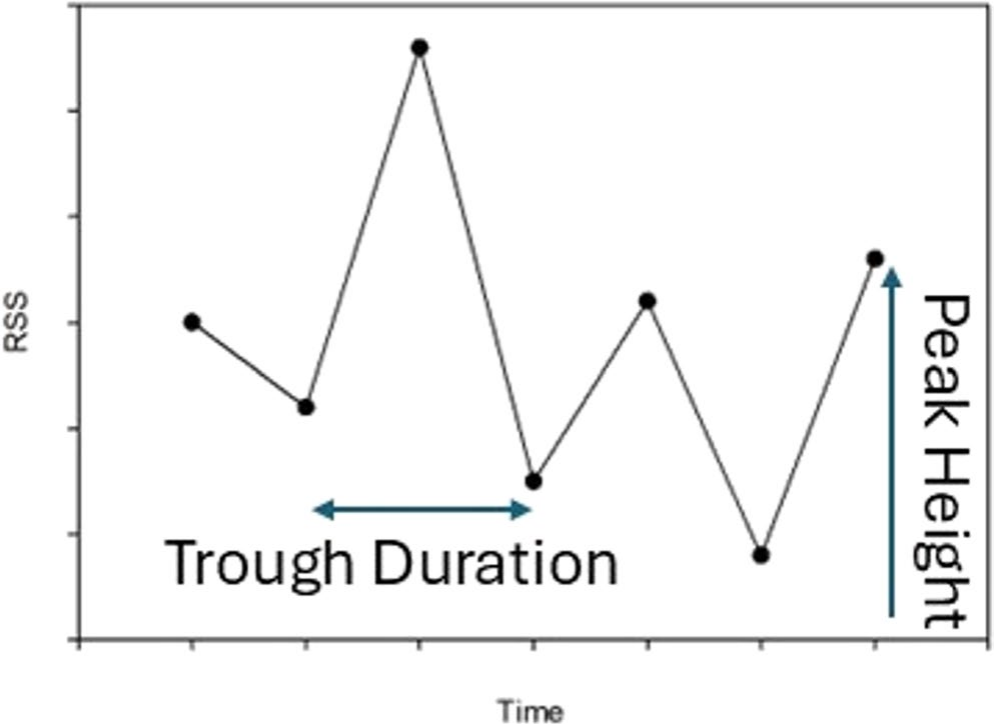
Trough duration and peak height calculation

In the resulting Root Sum Squares time series, trough-to-trough durations and peak heights were assessed in multiple ways for each participant. In the Esfahlani et al. (2022) study, a trough was defined as a value that was lower than the values directly before and after it in the RSS time series, and a peak was defined as the high value between two troughs as shown in Fig. 1.

These definitions of trough duration and peak height were used in the initial analysis, but in some cases a trough point was minimally different than the preceding or successive data point. Therefore, additional analyses were performed in which the trough point had to be either 5, 2, or 1% lower than the preceding and successive critical values. Peak height was analyzed based on the y axis value for the peak. The range of values in the RSS series in the current study is greater than the data range found in the Esfahlani et al. (2022) study due to the fact that the present study utilized a larger number of nodes, resulting in a larger number of edges. Therefore, edge RSS values in the current study would be correspondingly higher.

S/g effects for trough duration and peak height were assessed using IBM SPSS Statistics Version 29 general linear model (GLM) analyses with gender as the independent variable and total brain volume and age as covariates for each definition of troughs. Total brain volume was computed as the sum of gray matter and white matter shown in the HCP Freesurfer data.

## Results

**Fig. 2:**
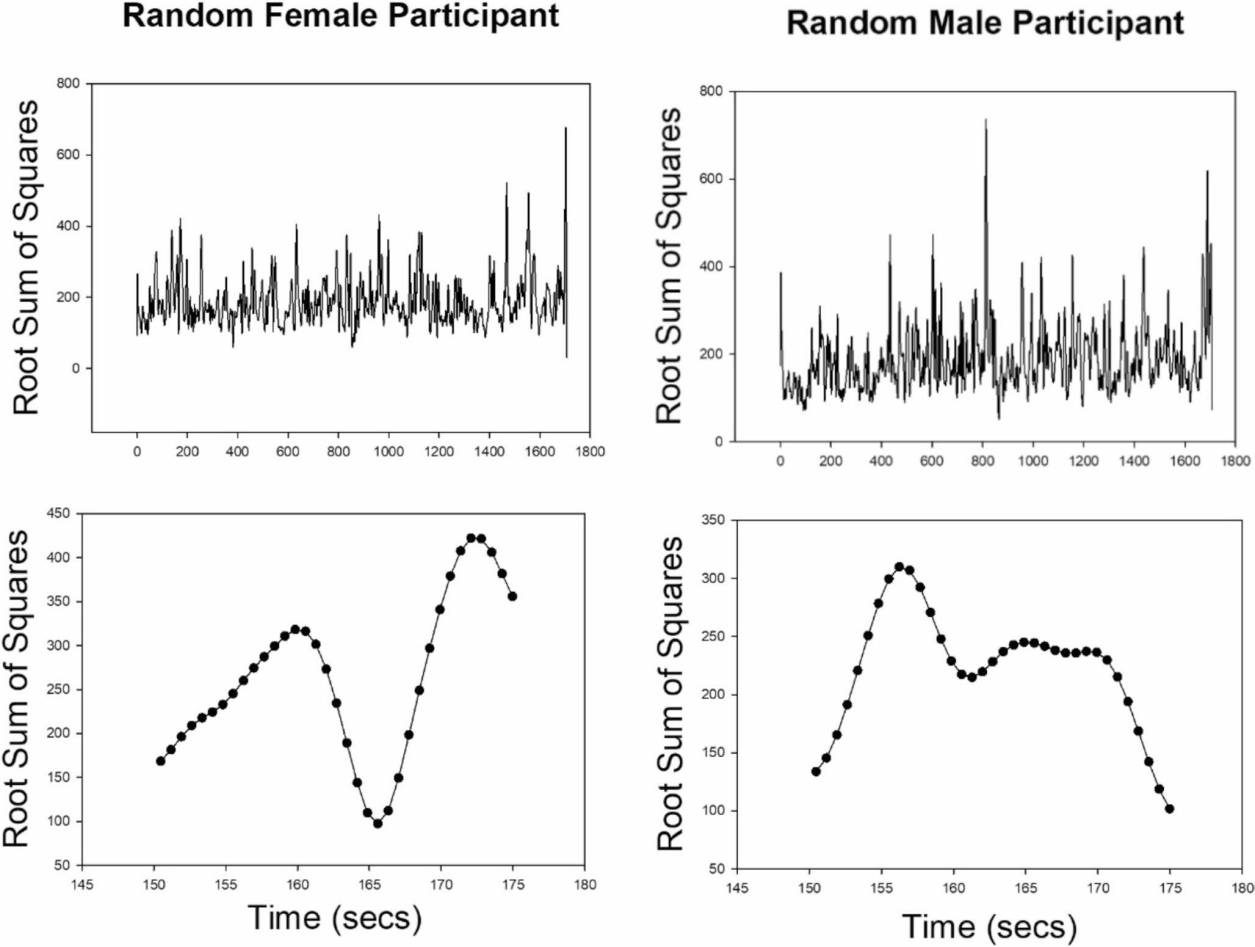
Whole-brain RSS plots for full scan duration and random 25 s interval for a random male and female participant

Plots of the RSS time series for a randomly selected male and female participant are shown in Fig. 2, along with enlarged segments shown for a 25 s interval.

### S/G whole brain effects

**Fig. 3:**
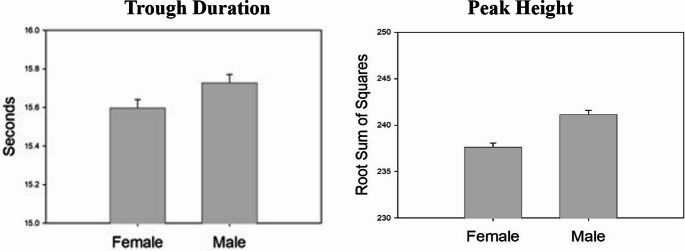
Estimated mean (SE) of female and male trough duration and peak height with troughs defined as any points lower than preceding and successive values

Using the definition of a trough as any point lower than its preceding and successive value in the RSS time series, with total brain volume and age as covariates, males had longer trough durations (*F* (1,1020) = 5.95, *p* <.02). Males also showed greater peak heights *F* (1,1020) = 32.55, *p* <.001). These effects are shown in Fig. 3.

Additional analyses were conducted using the criterion that a trough had to be either 1, 2, or 5% different in magnitude from the preceding and subsequent critical values. The resulting trough durations and peak heights are shown in Table 2.

**Table 2:** Estimated Mean (SE) Trough Durations and Peak Heights as a Function of Sex/Gender Based on Trough Definition. Trough points required to be either 1, 2, or 5% different from preceding and successive points

Magnitude of change required for trough				
Whole Brain Analysis				
	> 0 %	1 %	2 %	5 %
Trough Duration (seconds)				
Male	see Figure 3	16.6 (.04)	17.1 (.05)	18.4 (.05)
Female		16.4 (.04)	16.8 (.04)	18.0 (.05)
Trough Duration (seconds)				
Male		235.6 (.30)	237.6 (.30)	242.1 (.32)
DMN Analysis				
	> 0 %	1 %	2 %	5 %
Trough Duration (seconds)				
Male	15.7 (.04)	16.3 (.04)	16.7 (.04)	17.6 (.05)
Female	15.5 (.04)	16.0 (.04)	16.4 (.04)	17.2 (.04)
Peak Height (RSS value)				
Male	45.8 (.06)	46.4 (.06)	46.6 (.06)	47.6 (06)
Female	46.3 (.05)	46.8 (.06)	47.1(.06)	48.0 (.06)

These analyses revealed consistent s/g effects in trough duration, but not in peak height, with age and total brain volume as covariates. Using the 1% criterion, trough duration was significantly longer for males than females, *F* (1,1020) = 13.48, *p.* < 0.001, but peak height was greater for females, *F* (1,1020) = 4.79, *p.* < 0.02. At the 2% criterion, trough duration again was significantly longer for males, *F* (1,1020) = 18.71, *p.* < 0.001, while peak height was marginally higher for females, *F* (1,1020) = 3.05, *p. <* 0.10). At the 5% criterion, trough duration remained significantly longer for males than females, *F* (1,1020) = 23.34, *p.* < 0.001, but peak height did not differ.

### S/G DMN effects

Parallel analyses using data from only the DMN found similar effects for trough duration, but a clearer pattern of peak height differences. The data are shown in Table 2. Males had significantly longer trough durations at each definition of trough (> 0, 1, 2 or 5% change): *F* (1,1200) = 7.25, *p.* < 0.01 at 0% change, *F* (1,1200) = 15.15, *p.* < 0.001 at 1%, *F* (1,1200) = 20.28, *p* <.001, and *F* (1,1200) = 29.13, *p* <.001 at 5%. Females showed significantly greater peak heights in all 4 conditions (*F* (1,1200) ≥ 19.55, *p* <.001).

## Discussion

To our knowledge, these analyses are the first to suggest possible s/g differences in dynamic functional connectivity based on edge analyses. In this relatively large sample, males consistently showed longer trough durations across all of the analyses. This effect was small but statistically significant in all cases, and was obtained while controlling for brain volume. Peak height effects were more variable and changed as a function of how troughs were defined in the whole brain analysis, but females showed consistently greater peak height in the DMN analysis. Given the consistency of the trough duration effects, this seems to represent a genuine s/g difference that merits further investigation. The finding of greater peak heights in the DMN for females may be consistent with node based FC analyses that find greater connectivity within the DMN (de Lacy et al., 2019).

While it is tempting to speculate about what these findings mean, it would be premature to draw any strong conclusions. In their study contrasting RSS peaks and troughs between ASD individuals and controls based on data obtained while participants watched movies, Esfahlani et al. (2022) observed that ASD individuals had longer trough durations, but no difference in peak heights, and suggested that network dynamics might be “stickier” in the ASD group. In the present case, one could either suggest that males also show “stickier” network dynamics, or conversely that females show a greater flexibility in network dynamics. Understanding the significance of these effects awaits further investigations relating RSS values to other factors such as behavioral characteristics. Notably, the present results were obtained from participants in a resting state condition, while the results from that study reflected participants viewing movies. One extension of the current work would be to ascertain whether the observed s/g differences persist when participants are in various task states.

Another possible extension of the current approach would be to continue to explore brain network dynamics examining subnetworks in addition to the DMN. The whole brain analysis is appealing in its simplicity, but it leaves open the possibility that many more interesting changes in dynamic connectivity are occurring in subnetworks but go undetected when the data is combined into a single metric. The finding of greater peak heights for females in the DMN analysis, but not consistently in the whole brain analysis, is consistent with this possibility.

## Conclusions

Overall, the present findings indicate a novel s/g difference in whole brain dynamic functional connectivity in which male brains reconfigure less frequently than female brains in resting state. In the DMN, males again showed less frequent reconfigurations while females showed greater peak heights. Additionally, they provide further evidence of the utility of the RSS procedure for examining group and individual differences.

## Data Availability

Minimally preprocessed fMRI data was obtained from the Human Connectome Project Young Adult 1200 Subjects Data Release. Additional data is available from the authors on request.

## Code Availability

Matlab code for the RSS analyses is available at https://github.com/rlyday/LCBN_RSS.
